# Characterization and engineering of broadly reactive monoclonal antibody against hepatitis B virus X protein that blocks its interaction with DDB1

**DOI:** 10.1038/s41598-019-56819-8

**Published:** 2019-12-30

**Authors:** Shuai Tao, Shaokun Pan, Chenjian Gu, Lili Wei, Ning Kang, Youhua Xie, Jing Liu

**Affiliations:** 1 Key Laboratory of Medical Molecular Virology (MOE/NHC/CAMS), Department of Microbiology and Parasitology, School of Basic Medical Sciences, Shanghai Medical College, Fudan University, Shanghai, China; 2 National Clinical Research Center for Aging and Medicine, Huashan Hospital, Fudan University, Shanghai, China; 3 Key Laboratory of Medical Epigenetics and Metabolism, Institutes of Biomedical Sciences, School of Basic Medical Sciences, Shanghai Medical College, Fudan University, Shanghai, China

**Keywords:** Applied immunology, Protein delivery, Hepatitis B virus, Virus-host interactions

## Abstract

Hepatitis B virus (HBV) X protein (HBx) plays diverse roles in both viral life cycle and HBV-related carcinogenesis. Its interaction with DNA damage-binding protein 1 (DDB1) was shown to be essential for engendering cellular conditions favorable for optimal viral transcription and replication. Previously, we described a mouse monoclonal antibody against HBx (anti-HBx 2A7) recognizing HBx encoded by representative strains from 7 of 8 known HBV genotypes. In this work, we further characterized 2A7 in order to explore its potential usefulness in HBx-targeting applications. We demonstrated that 2A7 recognizes a linear epitope mapped to L^89^PKVLHKR^96^ on HBx, a segment that is highly conserved across genotypes and coincidentally overlaps with the DDB1-interacting segment. HBx-DDB1 binding could be inhibited by 2A7 *in vitro*, suggesting therapeutic potential. Nucleic acid and amino acid sequences of 2A7 were then obtained, which allowed construction of recombinant antibody and single chain variable fragments (scFv). 2A7-derived recombinant antibody and scFv recapitulate 2A7’s HBx-binding capacity and epitope specificity. We also reported preliminary results using cell-penetrating peptide for delivering 2A7 antibody across cell membrane to target intracellular HBx. Anti-HBx 2A7 and 2A7-derived scFv characterized here may give rise to novel HBx-targeting diagnostics and therapeutics for HBV- and HBx-related pathologies.

## Introduction

Hepatitis B virus (HBV) is the type member of *Hepadnaviridae*, a family of small, non-cytopathic viruses with a relaxed circular DNA (rcDNA) genome. Upon infection, the rcDNA genome is converted into covalently closed circular DNA (cccDNA) in the nucleus of infected cells^1^. HBV takes human as its only natural host and almost exclusively infects hepatocytes^2,3^. HBV infection usually causes either subclinical or symptomatic acute hepatitis, with the latter developing into chronic infection in a majority of infected neonates and young children, and a small percentage of adults^4^. Chronic HBV infection generally persists for life and has a high risk for progressing onto liver fibrosis, cirrhosis and eventually, hepatocellular carcinoma (HCC)^5,6^.

HBV genome contains 4 overlapping open reading frames (ORFs) that encode 7 viral proteins through the use of alternative start codons^1^. HBV X (HBx) is the smallest (154 amino acid residues) of HBV-encoded proteins and is found in all mammalian hepadnaviruses^7^. HBx is generally believed to be a non-structural protein and has been shown to play diverse functions in viral life cycle, virus-cell interactions and HBV-related HCC^7–10^. For instance, HBx positively contributes towards HBV transcription and replication both *in vitro* and *in vivo*^7,11–13^. In hepatoma cells, HBx is apparently involved with various key signaling pathways including Wnt/β-catenin^14^, NF-κB^15^, Notch^16^, RIG-I^17^, RAR/RXR^18^, *etc*., which underlies its association with HBV-related carcinogenesis.

Although multiple studies point to HBx as a regulator of viral and host gene expression, it has not been demonstrated to bind HBV or cellular DNA directly. Instead, mechanistic studies have shown that HBx acts through interacting with cellular adaptor proteins^19–21^ and DDB1 (DNA damage-binding protein 1) is one of the earliest HBx-binding host protein identified^19^. DDB1 is part of the DDB1-containing E3 ubiquitin ligase (also called CUL4-DDB1 or DDB1-CUL4 ubiquitin ligase) complex. Through its interaction with other adaptor proteins, DDB1 recruits substrates to the complex for ubiquitination and eventual proteasomal degradation^22,23^. HBx-DDB1 interaction has been shown to subvert this process in the virus’s favor, leading to enhanced cccDNA transcription and viral replication^24–26^. The fact that HBx requires interaction with cellular adaptor proteins to be functional suggest the possibility of targeting such interactions for treating liver diseases associated with HBV or HBx.

Previously, we described a broadly reactive mouse monoclonal antibody against HBx (anti-HBx 2A7) that detects HBx encoded by representative strains from 7 of 8 known HBV genotypes in Western blot and immunofluorescence assays^27^. In this work, mechanism(s) underlying 2A7’s broad reactivity and its potential usefulness in HBx-targeting applications are explored. We demonstrated that 2A7 recognizes a linear epitope encompassing amino acids 89 to 96 of HBx, which is highly conserved across genotypes and coincidentally overlaps with HBx’s DDB1-interacting segment. *In vitro*, binding between HBx and DDB1 was inhibited in the presence of 2A7. Complete nucleic acid and amino acid sequences of 2A7 were then obtained and used for the construction of recombinant antibody and single chain variable fragments (scFv). We show that 2A7-derived recombinant antibody and scFv recapitulates 2A7’s HBx-binding capacity and epitope specificity. Furthermore, we explored the possibility of delivering 2A7 into intracellular space for potential HBx-targeting applications.

## Results

### Monoclonal anti-HBx 2A7 recognizes native and denatured HBx proteins with broad reactivity

**Figure 1:**
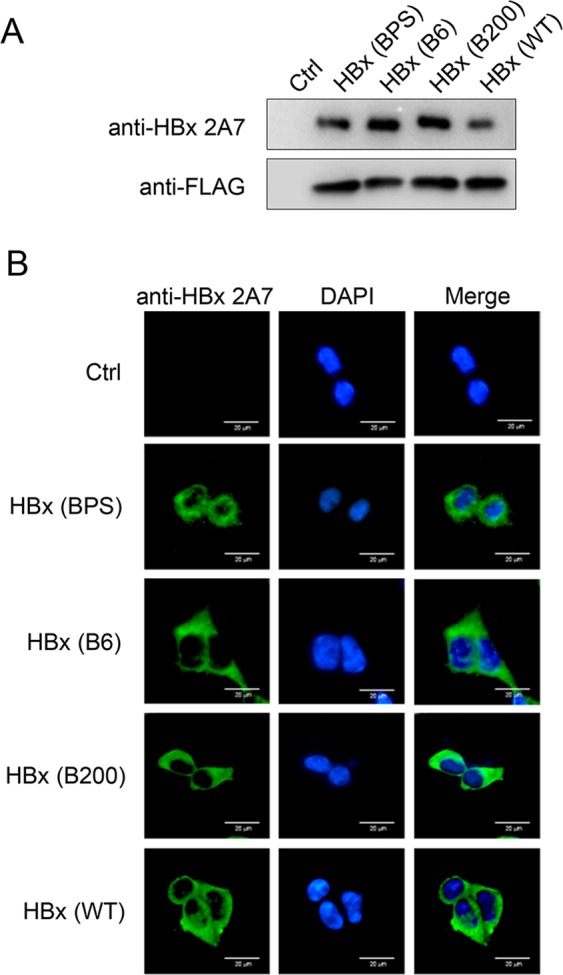
Anti-HBx mAb 2A7 broadly reacts with native and denatured HBx encoded by multiple HBV strains. HEK293T cells were transfected with plasmids expressing indicated HBx variants fused to N-terminal FLAG tag, and 48 hours later, were analyzed in Western blot (**A**) and immunofluorescence (**B**) using monoclonal anti-HBx antibody 2A7. FLAG antibody was used as control in Western blot (**A**). Cell nuclei were stained using DAPI (**B**). Scale bars, 20 μm.

In order to probe potential applications of monoclonal anti-HBx antibody 2A7, we first reconfirmed its reactivity and specificity in both Western blot and immunofluorescence. Three genotype B (BPS, B6 and B200) and one genotype C (WT) HBV isolates were used. BPS is the isolate from which the HBx immunogen used for preparing 2A7 was derived. HBx from these isolates display varying differences at protein sequence level (Supplementary Fig. S1). As shown in Fig. 1, 2A7 specifically recognizes all four HBx proteins expressed in HEK293T cells in both Western blot and immunofluorescence, in agreement with our previous results demonstrating its broad reactivity against HBx encoded by representative strains of most known HBV genotypes^27^. In contrast, another monoclonal anti-HBx obtained in parallel to 2A7, designated 2A2, only reacted strongly with immunogen BPS HBx in Western blot, with minimal reactivity against B200 HBx and no reactivity against B6 or WT HBx (Supplementary Fig. S2A). In immunofluorescence, 2A2 displayed comparable reactivity against both BPS HBx and B200 HBx, with no recognition of B6 and WT HBx as in Western blot (Supplementary Fig. S2B).

### Anti-HBx 2A7 epitope is mapped to a highly conserved segment of HBx

**Figure 2:**
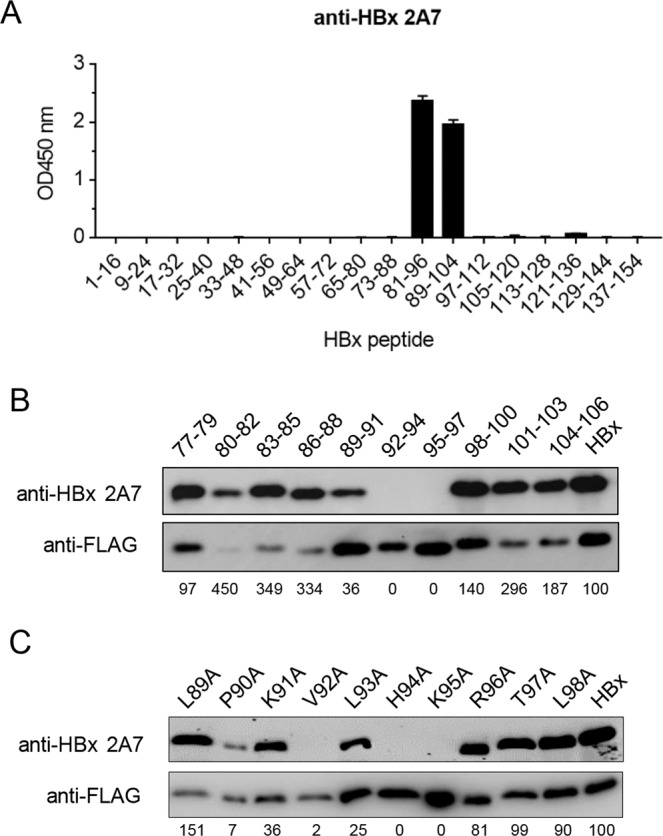
Epitope mapping of broadly reactive anti-HBx mAb 2A7. (**A**) Serially overlapping biotinylated peptides encompassing full BPS HBx length were captured onto Streptavidin-coated plates and subjected to ELISA using 2A7. Optical densities at 450 nm (OD_450_) were measured and plotted. Start and end positions of each peptide are indicated. (**B**) Plasmids expressing FLAG-tagged BPS HBx with serial 3 amino acid residues mutated to 3 Ala as indicated were transfected into HEK293T. (**C**) Plasmids expressing FLAG-tagged BPS HBx with indicated single residue mutation were transfected into HEK293T. Transfected cells were analyzed in Western blot using 2A7 and FLAG antibody. Densitometry scanning of the blots was performed, and 2A7-generated signals were first adjusted against anti-FLAG-generated signals from the same sample, followed by normalization against BPS HBx. Normalized values are indicated as percentages under each lane.

In an attempt to establish the molecular basis of 2A7’s broad reactivity, we mapped its epitope first by using a series of overlapping 16 amino acid (a.a.) peptides that encompass full-length BPS HBx. Only two peptides, harboring a.a. 81–96 and 89–104 respectively, were recognized by 2A7 in ELISA (Fig. 2A), indicating that the 2A7 epitope is localized between a.a. 81–104, with a.a. 89–96 (LPKVLHKR) being the essential part.

More precise mapping was then performed by consecutively mutating 3 a.a. to alanine residues within a.a. 77–106 of BPS HBx. Western blot using 2A7 against the mutants showed that mutations of a.a. 92–94 and 95–97 abrogated recognition by 2A7, and mutation of a.a. 89–91 also resulted in markedly decreased reactivity with 2A7, whereas other mutants retained full or nearly full 2A7 reactivity (Fig. 2B). This result is in agreement with data obtained using overlapping peptides and reconfirmed that a.a. 92–96, and a.a. 89–91 to a lesser extent, are essential for 2A7 recognition.

Furthermore, the importance of each residue within 2A7 epitope a.a. 89–96 was analyzed using single residue alanine scanning. As shown in Fig. 2C, the most significant effect was observed for P90, V92, H94 and K95 where mutation to alanine abrogated 2A7 reactivity. Mutation to alanine at K91 and L93 also negatively impacted reactivity, while mutation of other tested sites showed relatively minor or no effects on 2A7 binding. These data are consistent with results shown above, and identified key residues within the a.a. 89–96 epitope that play vital roles in interactions with 2A7.

**Figure 3:**
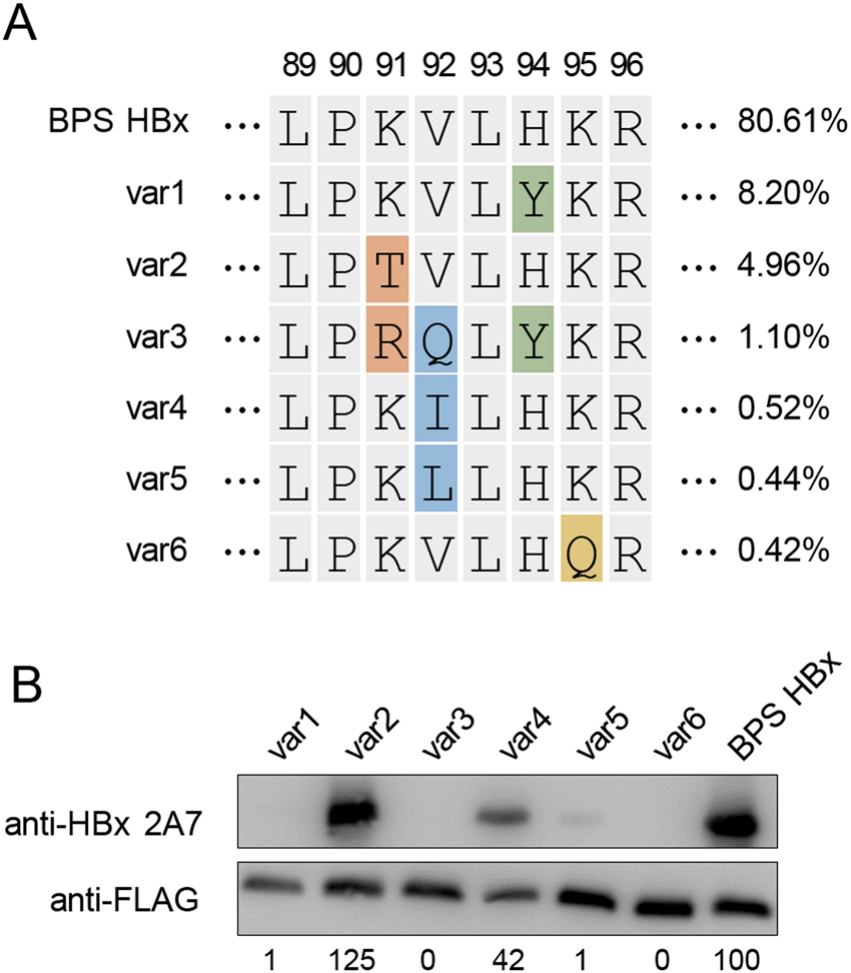
Anti-HBx mAb 2A7 epitope is mapped to a highly conserved region of HBx. (**A**) Alignment of most frequently found types of sequences among ~7000 full-length HBx sequences retrieved from GenBank at 2A7 epitope (a.a. 89–96). Colored residues highlight differences from BPS HBx. Frequency of each variant is indicated. (**B**) Plasmids expressing FLAG-tagged BPS HBx with a.a. 89–96 mutated to sequences of indicated variant as shown in (**A**) were transfected into HEK293T cells. Transfected cells were analyzed in Western blot using 2A7 and FLAG antibody. Densitometry scanning of the blots was performed, and 2A7-generated signals were first adjusted against anti-FLAG-generated signals from the same sample, followed by normalization against BPS HBx. Normalized values were indicated as percentages under each lane.

After mapping the essential 2A7 epitope on HBx to a.a. 89–96, HBx sequences used in Fig. 1 were checked and, unsurprisingly, HBx encoded by the three non-BPS strains have exactly the same a.a. sequences at this site compared to immunogen BPS HBx (Supplementary Fig. S1). In order to evaluate the conservedness of HBx sequences at 2A7 epitope among known HBV variants, ~7000 full-length HBx protein sequences were retrieved from GenBank and aligned against BPS HBx. The alignment showed that 80.61% sequences are identical with BPS HBx at a.a. 89–96 (Fig. 3A), indicating that 2A7 epitope lies within a highly conserved segment. Indeed, on HBV genome, this segment is located within the N-terminus of the overlapping enhancer II regulatory element^28^, which is essential for liver-specific transcription of HBV promoters^1^. Moreover, even within the remaining ~20% sequences, variations within this segment are fairly limited. When the six most common naturally occurring variations compared to BPS HBx, namely H94Y (8.20%), K91T (4.96%), K91R/V92Q/H94Y (1.10%), V92I (0.52%), V92L (0.44%) and K95Q (0.42%) were introduced into BPS HBx and tested for 2A7 recognition in Western blot, K91T and V92I mutants retained full and nearly half 2A7 reactivity, respectively, while the other mutants were not efficiently detected by 2A7 (Fig. 3B).

Combined with results from single residue alanine scanning, these data suggested the importance of key residues within 2A7 epitope for optimal reactivity. H94 and K95 appear to be critical, because both artificial H94A and K95A mutations and naturally occurring H94Y and K95Q variations would abrogate 2A7 binding (Figs. 2C and 3B). K91, on the other hand, can tolerate K91T variation without losing 2A7 reactivity, but K91A mutation markedly affects 2A7 recognition. In contrast, V92 cannot tolerate mutation to alanine, and recognition by 2A7 of naturally occurring variation V92L was also severely affected. Variant V92I, however, can be recognized by 2A7, despite being highly similar to the none-reactive V92L variant.

Clearly, 2A7 binding to HBx involves interactions with multiple residues within a.a. 89–96, with different requirements for each position for maximal reactivity. Data presented in Fig. 3 demonstrate that despite non-recognition of fairly common variants like H94Y (8.20%), 2A7 collectively recognizes at least 86% (canonical 80.61%, K91T 4.96%, V92I 0.52%) of known HBx variants. Such broad reactivity makes 2A7 highly promising for potential diagnostic and therapeutic applications targeting HBx.

Similar epitope mapping workflow was applied to 2A2 and its epitope was roughly mapped to a.a. 17–32, a segment that harbors significant variations at the C-terminal half (Supplementary Fig. S3), even between the three non-BPS strains used in Fig. 1 (Supplementary Fig. S1).

### Anti-HBx 2A7 epitope coincides with DDB1 binding site allowing 2A7 to block HBx-DDB1 interaction

**Figure 4:**
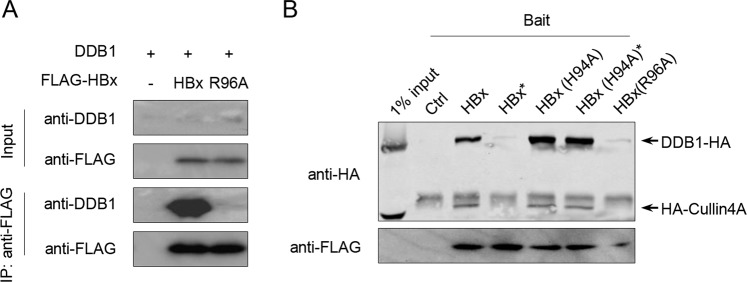
Anti-HBx mAb 2A7 specifically inhibits HBx-DDB1 interaction. (**A**) Plasmids expressing FLAG-tagged wild type BPS HBx or HBx (R96A) mutant were co-transfected with DDB1 expression plasmid into HEK293T cells. Transfected cells were lysed and subjected to co-immunoprecipitation using anti-FLAG as capture antibody. Input and precipitated proteins were analyzed using anti-FLAG and anti-DDB1 antibodies as indicated. (**B**) HEK293T cells co-transfected with HA-tagged DDB1 and Cullin4A and cells transfected with wild type or mutant BPS HBx expression plasmids as indicated were used to prepare cell lysates. DDB1-HA/HA-Cullin4A-containing lysates were then mixed with FLAG-HBx-containing lysates with (indicated by *) or without addition of 2A7 antibody, and subjected to pull-down using anti-FLAG antibody. Captured proteins were analyzed using anti-FLAG and anti-HA antibodies.

Previous studies of HBx functions have identified DDB1 as a cellular protein that binds HBx, and DDB1-HBx interactions have been shown to subvert normal functions of CUL4-DDB1 ubiquitination machinery to favor optimal HBV replication^24,25^. HBx interacts with DDB1 through a short segment encompassing a.a. 88–100^29^, which overlaps with 2A7 epitope a.a. 89–96, making 2A7 a potential competitive inhibitor of HBx’s interactions with DDB1. To test such a possibility, we first reconfirmed the binding between HBx and DDB1 in co-immunoprecipitation assay and the lack of interaction between DDB1 and HBx mutant R96A, which has been reported to be incompetent for DDB1-binding^29^ (Fig. 4A). FLAG-tagged wild type HBx or HBx mutants, and HA-tagged DDB1 plus HA-tagged Cullin4A were then subjected to pull-down assay using anti-FLAG antibody in the presence or absence of 2A7 antibody. As shown in Fig. 4B, pull-down of DDB1/Cullin4A by FLAG-HBx (wild type) was markedly inhibited by addition of 2A7 antibody. On the other hand, a mutant HBx harboring a single amino acid mutation (H94A) that does not affect DDB1-binding^29^ but prevents 2A7 recognition (Fig. 2) could pull down DDB1/Cullin4A regardless of the presence or absence of 2A7 antibody. In contrast, 2A2, which recognizes an epitope distant from DDB1-interacting region (Supplementary Fig. S3), had no such effect (Supplementary Fig. S4). These results indicate that the coincidental overlapping of 2A7 epitope and DDB1-binding site on HBx indeed enables 2A7 to block HBx-DDB1 binding and point to the potential usefulness of 2A7 in HBx targeting applications.

### Determination of 2A7 amino acid sequences using combined RT-PCR and mass spectrometry

Since the broadly-active anti-HBx mAb 2A7 showed potential as HBx-targeting agent, we attempted to determine its amino acid sequences in order to allow further engineering. RNA was extracted from the hybridoma cell line producing 2A7 and subjected to RT-PCR using a panel of primer sets designed for amplification of cDNA encoding mouse IgG constant and variable regions^30^. Amplicons with expected length were cloned into TA-vector and multiple randomly selected clones were sequenced to avoid missing potential heterogeneity. De-duplicated sequencing results (Supplementary Fig. S5 & S6) showed that for variable regions, only one sequence (2A7 VH) was obtained for heavy chain, while two different sequences were obtained for light chain: 2A7 VL1 and VL2term, with the latter containing an internal termination codon; for constant regions, only one sequence (2A7 CH) was obtained for heavy chain, and three different sequences were obtained for light chain: one κ-type sequence (2A7 CK), and two highly similar λ-type sequences (2A7 CL1 and CL2).

In order to obtain 2A7 amino acid sequences, purified 2A7 antibody protein was subjected to reductive SDS-PAGE followed by in-gel trypsin digestion and LC-MS/MS analysis. Raw data from MS were then analyzed in MaxQuant software using candidate cDNA sequences obtained above as queries. As shown in Supplementary Table S8, 2A7 VL1/CK and VH/CH encoded peptides account for the predominant majority of MS signals derived from trypsin-digested 2A7 light and heavy chains respectively, confirming that these sequences indeed represent cDNA sequences for 2A7. Using BLAST software, 2A7 CH was mapped to IGHG1 (immunoglobulin heavy constant gamma 1) gene on mouse genome, indicating that 2A7 mAb is of IgG1 subclass.

### Recombinant 2A7 antibody and 2A7-derived scFv display identical epitope-specificity as 2A7 mAb

**Figure 5:**
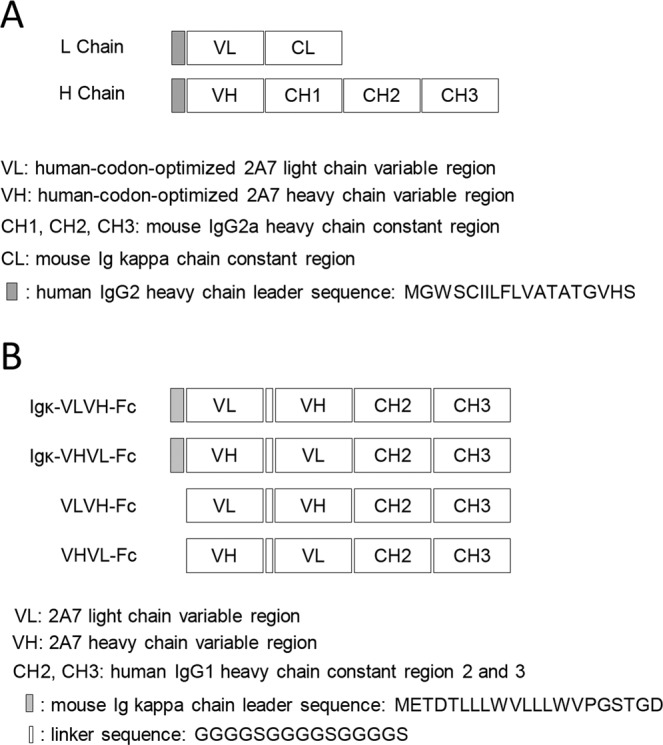
Schematic representation of 2A7-derived recombinant antibody and scFv constructs. Configurations designed to express recombinant 2A7 heavy and light chains (**A**), and secreted or non-secreted 2A7-derived scFv constructs (**B**) are shown.

**Figure 6:**
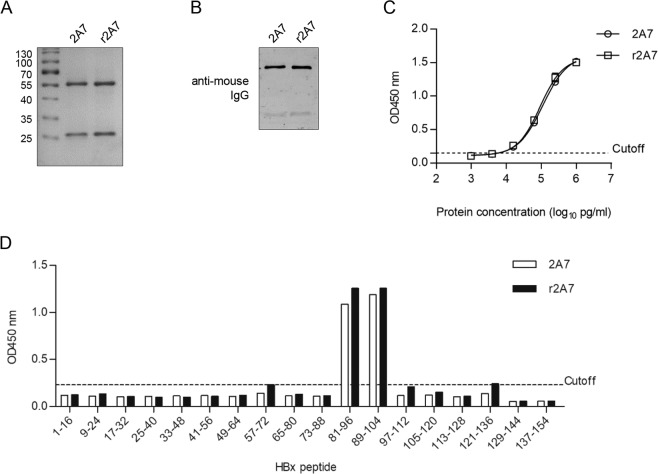
Recombinant 2A7 reproduces 2A7’s HBx reactivity and epitope specificity. Recombinant 2A7 antibody was purified from culture supernatants of Expi293F^TM^ cells co-transfected with recombinant heavy chain and light chain expression plasmids (see Fig. 5A), and analyzed in SDS-PAGE (**A**), Western blot (**B**), BPS HBx ELISA (**C**) and epitope-mapping assay as shown in Fig. 2A (**D**). 2 A7 antibody was used in parallel in all the assays.

In order to further confirm the correctness of 2A7 variable region sequences obtained above, human-codon-optimized 2A7 heavy and light chain variable region coding sequences were cloned between N-terminal human IgG2 heavy chain secretion signal and C-terminal mouse IgG2a heavy chain and κ light chain constant regions, respectively (Fig. 5A), and then co-transfected into Expi293F^TM^ cells. Recombinant 2A7 secreted into culture supernatant (Fig. 6A,B) were enriched and purified using protein A agarose and analyzed for reactivity with HBx protein. Compared to the original hybridoma-derived 2A7, recombinant 2A7 displayed similar reactivity with HBx in ELISA (Fig. 6C) and epitope-mapping assay confirmed that recombinant 2A7 specifically recognizes the same epitope (Fig. 6D). These data unequivocally proved that amino acid sequences of 2A7 variable regions were correctly obtained, and 2A7 reactivity and specificity can be fully recapitulated using recombinant constructs.

**Figure 7:**
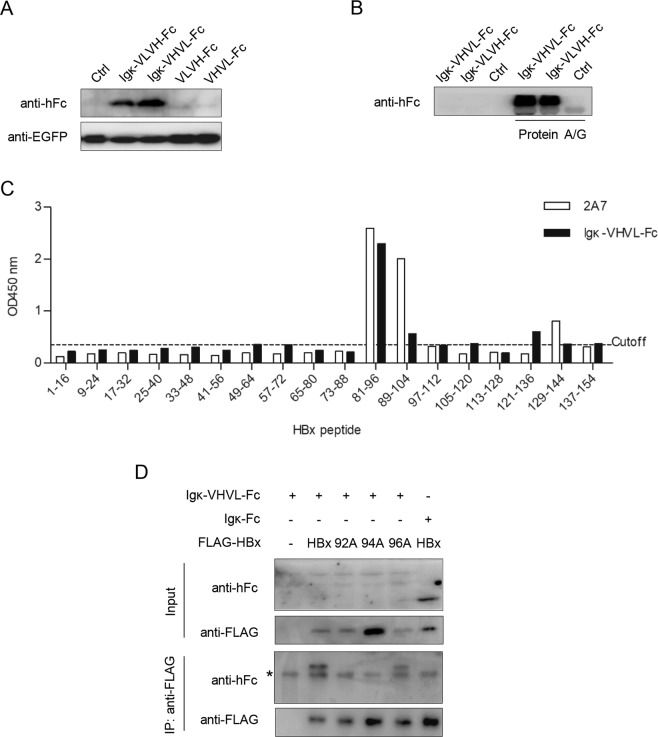
2A7-derived scFv constructs reproduce 2A7’s HBx reactivity and epitope specificity. (**A**) HEK293T cells transfected with expression plasmids of indicated 2A7 scFv constructs (see Fig. 5B) were subjected to Western blot analysis using anti-hFc antibody. Cells were co-transfected with EGFP expression plasmid as transfection control and EGFP in the same cell lysate samples were detected using anti-EGFP. (**B**) Supernatants of cells transfected with Igκ-VLVH-Fc or Igκ-VHVL-Fc were subjected to Western blot analysis using anti-hFc antibody with or without prior enrichment using protein A/G agarose. (**C**) Epitope-mapping assay as shown in Fig. 2A was performed using secreted Igκ-VHVL-Fc and 2A7 as control. (**D**) HEK293T cells were co-transfected with expression plasmids of Igκ-VHVL-Fc and indicated FLAG-tagged wild type or mutant BPS HBx. Cell lysates were subjected to co-immunoprecipitation assay using anti-FLAG as capture antibody. Igκ-VHVL-Fc and HBx variants in input and captured fractions were detected using anti-hFc and anti-FLAG antibodies, respectively. *, non-specific signal produced by anti-hFc antibody.

Next, we constructed a series of scFv configurations using 2A7 variable regions (VH and VL) concatenated in reciprocal order and fused at C-terminal to human IgG1 Fc, with or without N-terminal mouse Igκ secretion signal sequences (Fig. 5B). Upon transfection of HEK293T cells, both Igκ-VLVH(2A7)-Fc and Igκ-VHVL(2A7)-Fc displayed prominent expression (Fig. 7A) and secretion (Fig. 7B), whereas constructs lacking secretion signals showed minimal expression. The efficiently expressed Igκ-VHVL(2A7)-Fc (Fig. 7A) was selected for detailed characterization. Epitope mapping confirmed that Igκ-VHVL(2A7)-Fc recognized the same epitope as 2A7 (Fig. 7C). Moreover, in pull-down assay, Igκ-VLVH(2A7)-Fc interacted with wild-type and R96A mutant HBx, but not V92A or H94A mutants (Fig. 7D), which echoed 2A7’s reactivity with these mutants (Fig. 2C). Taken together, these results confirmed that 2A7-derived scFv Igκ-VLVH(2A7)-Fc recapitulates the specific epitope-reactivity of the original 2A7 antibody.

### Epitope peptide fused with HIV Tat delivered 2A7 mAb into target cells while retaining HBx reactivity

**Figure 8:**
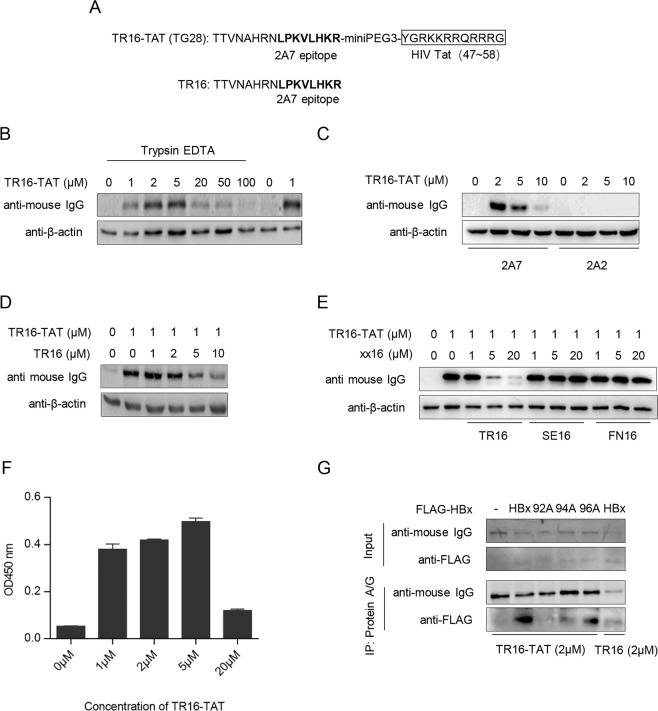
Delivery of 2A7 into intracellular space using CPP-tagged epitope peptide. (**A**) Design of CPP-tagged 2A7 epitope peptide (TR16-Tat): a.a. 81–96 of BPS HBx, which harbors 2A7 epitope a.a. 89–96, is fused with CPP of HIV-1 Tat (a.a. 47–58) through an intervening miniPEG3 linker. a.a. 81–96 of BPS HBx (TR16) without Tat CPP is used as control. (**B**) 150 μg/ml of 2A7 mAb was incubated with indicated concentrations of TR16-Tat in culture media at 37 °C for 30 minutes before being applied to Huh-7 cells. Treated cells were cultured for 6 hours and harvested for Western blot analysis, with or without Trypsin/EDTA treatment prior to lysing by SDS-PAGE loading buffer. Cell-associated 2A7 was detected using anti-mouse IgG and β-actin was detected in parallel as loading control. (**C**) 150 μg/ml of 2A7 or 2A2 were similarly incubated with indicated concentrations of TR16-Tat, applied to Huh-7 cells, and analyzed with Trypsin/EDTA pretreatment as shown in (**B**). (**D**,**E**), TR16 and neighboring overlapping HBx peptides SE16 (a.a. 65–80) and FN16 (a.a. 73–88) without CPP tags were added at indicated concentrations during pre-incubation as competitors, before TR16-Tat/2A7 was applied to Huh-7 cells and analyzed as in (**B**). (**F**) Huh-7 cells treated with 150 μg/ml of 2A7 pre-incubated with indicated concentrations of TR16-Tat were subjected to Trypsin/EDTA treatment followed by lysis in RIPA buffer. Intracellular 2A7 thus recovered was then detected in ELISA using BPS HBx coated microplate. (**G**) HepG2 cells were transfected with expression plasmids of indicated FLAG-tagged HBx variants and 36 hours later, cells were treated with 150 μg/ml of 2A7 pre-incubated with 2 μmol/L TR16-Tat or TR16, followed by co-immunoprecipitation assay using Protein A/G agarose. HBx and 2A7 in input and captured fractions were detected using anti-FLAG and anti-mouse IgG, respectively.

Despite its nearly identical epitope-specificity compared to 2A7, scFv VHVL(2A7) is only expressed at fairly low levels in a secretion-dependent manner (Fig. 7A,B). Since HBx does not contain secretion signal sequences and consequently localizes to and functions in intracellular space, it is unlikely that Igκ-VHVL(2A7)-Fc expressed in HBx-expressing cells would have the opportunity to encounter and interact with HBx. Interactions between Igκ-VHVL(2A7)-Fc and co-expressed HBx observed in pull-down assays (Fig. 7D) were therefore most likely enabled by the cell lysis step in the procedure. These issues negatively affect development of Igκ-VHVL(2A7)-Fc-based HBx-targeting applications. As an alternative, the possibility of delivering extracellular 2A7 antibody into intracellular space through the use of cell-penetrating peptides (CPP) was tested. We first fused recombinant 2A7 or its derivative scFv covalently with HIV Tat CPP, but failed to obtain detectable entry of fusion proteins (data not shown). A fusion peptide (TR16-Tat) was then chemically synthesized that contains a segment of HBx (a.a. 81–96) harboring the 2A7 epitope at the N-terminal and CPP domain (a.a. 47–58) of HIV-1 Tat protein at the C-terminal, joined together through an miniPEG3 linker (Fig. 8A). Theoretically, this peptide could bind to 2A7 antibody and, through the action of Tat CPP, potentially enable the antibody-peptide complex to traverse cell membrane.

To test this hypothesis, TR16-Tat was first allowed to incubate with purified 2A7 antibody before being added to culture media of Huh-7 cells. After incubation in the presence of antibody and peptide mixture, cells were washed and treated with trypsin to remove surface-attached 2A7. Western blot analysis of trypsin-treated cells showed that pre-incubation with TR16-Tat enables 2A7, but not 2A2, to enter cells (Fig. 8B,C). The amount of cell-associated 2A7 antibody correlates with the amount of TR16-Tat used for pre-incubation up to about 5 μmol/L, beyond which higher TR16-Tat concentration resulted in lower association. In order to further confirm the specificity of TR16-Tat-mediated 2A7 entry into cells, increasing amounts of TR16 peptide lacking Tat CPP was added during pre-incubation as competitor, which dose-dependently decreased the amount of cell-associated 2A7, demonstrating that antibody-epitope interaction is required (Fig. 8D). In contrast, two non-2A7-epitope HBx peptides had no competitive effects (Fig. 8E). Finally, 2A7 antibody delivered by TR16-Tat into Huh-7 and HepG2 cells was tested for its reactivity against HBx. As shown in Fig. 8F, intracellular 2A7 antibody recovered from lysed Huh-7 cells treated with TR16-Tat/2A7 was capable of recognizing HBx protein in ELISA. HepG2 cells transfected with FLAG-tagged wild type or mutant HBx were also treated with TR16-Tat/2A7 followed by co-immunoprecipitation using Protein A/G to capture intracellular 2A7. As shown in Fig. 8G, intracellular 2A7 delivered by TR16-Tat/2A7 could specifically bind wild-type and R96A mutant HBx, but not mutants that severely affect reactivity with 2A7 (compare with Fig. 2C). These data suggest that although TR16-Tat binding would partially occupy epitope binding sites on 2A7, after entering cells via the action of Tat CPP, the antibody would still be able to recognize and bind HBx while retaining its epitope-specificity.

## Discussion

The vital role played by HBx in HBV life cycle have been repeatedly demonstrated over the years, both *in vitro* and *in vivo*^1,7^. HBx’s association with and contribution to HBV-related HCC are also supported by numerous studies, although the details of which still require further elaboration and confirmation^9^. HBx is thus an obvious target for developing drugs against HBV infection or HBV-related HCC. In this work, we report detailed characterizations of a mouse monoclonal HBx antibody (2A7) that is capable of recognizing a predominant majority of known HBx variants (Fig. 3). In addition, we obtained full amino acid sequences of 2A7 and generated recombinant antibody and scFv constructs that were demonstrated to recapitulate 2A7’s epitope specificity (Figs. 6 and 7). The original 2A7 mAb and its derivatives described here provide powerful tools for HBx-targeting basic and applied research. For instance, we have successfully utilized 2A7-derived scFv to demonstrate the effectiveness of a system developed for screening HBx-targeting molecules^31^.

Limited information is available regarding possible differences in functions between naturally occurring HBx variants encoded by different HBV genotypes and strains. Lack of usable antibody tools is a possible factor in this regard, especially for studies using whole HBV genomes, because overlapping of HBx ORF with other virus ORFs and regulatory elements prohibits fusing HBx with tags to facilitate detection. It is not surprising that the epitope recognized by the broadly-reactive 2A7 was mapped to a highly conserved region of HBx (Figs. 2 and 3), which falls within the N-terminus of HBV enhancer II^28^. Earlier work has also shown that this region contains a binding site for C/EBP (CCAAT/enhancer-binding proteins) and is involved in regulating liver-specific HBV transcription^32,33^. It is likely that variation at 2A7 epitope site on HBx is to some extent constrained by viral dependence on the presence, at nucleic acid level, of functional transcription factor binding site(s) for optimal transcription and subsequent replication. Regardless of the underlying mechanism(s), conservation of this site enables 2A7 to recognize more than 86% of reported HBx protein sequences and such a high level of broad reactivity no doubt establishes 2A7 and its derivatives as powerful tools for HBx studies.

Although interaction between HBx and DDB1 has long been known^19^, it was not until recent years that its functional significance in viral life cycle has been demonstrated^24–26^. Hijacking of the cellular CUL4-DDB1 E3 ligase by HBx caused abnormal ubiquitination and degradation of proteins such as Smc5/6^25,26^, which in turn resulted in enhanced transcription from episomal HBV cccDNA and viral replication^7,10^. The binding site for DDB1 on HBx coincidentally overlaps with 2A7 epitope and our results show that in the presence of 2A7, binding between HBx and DDB1 is largely inhibited (Fig. 4). This suggests the possibility of using 2A7 and its derivatives to target HBx-DDB1 interaction with the long-term aim of inhibiting HBV transcription and replication in infected hepatocytes. The fact that this segment of HBx is highly conserved (see above) further underlies the potential usefulness of this target and 2A7 as its blocker.

Since HBx is an intracellular protein, any potential HBx-targeting agents must first gain access to the intracellular space. Compared to IgG, recombinant scFv has much smaller gene and protein sizes, allowing easier manipulation and administration using both nucleic acid- and protein-based delivery methods. Among 2A7-derived scFv constructs that we tested, only those with leading secretion signal sequences were efficiently expressed (and secreted) in transfected cells (Fig. 7A,B), making it impossible to use 2A7-derived scFv as intrabody. The reason is not yet clear, but it is likely that entry into and transit through the secretion pathway, where chaperones and glycosidases localize and function, allow scFv to fold correctly. scFv without a secretion signal, on the other hand, may tend to misfold and end up being degraded in the cytoplasm. Since the secreted scFv was demonstrated to recapitulate 2A7 epitope specificity (Fig. 7), the possibility of a functional 2A7-derived scFv intrabody still warrants further study, *e.g*. through modifying scFv amino acid sequences and/or introducing co-expressed chaperone(s). If achievable, such an intrabody would enable HBx and HBx-DDB1 targeting applications upon liver-specific protein or gene delivery.

In addition to intrabody, delivery of protein across plasma membrane with the help of CPP is another possible method for targeting intracellular protein with specific antibody and derivatives. Our results using a fusion peptide combining HIV Tat CPP and 2A7 epitope showed that such a dual-function peptide could indeed bind 2A7 through the epitope and deliver the peptide-antibody complex into cells through the CPP (Fig. 8). Furthermore, intracellular 2A7 thus delivered retains the capability of recognizing and binding HBx (Fig. 8G). We believe that the bi-valent nature of IgG allows the epitope-CPP fusion peptide to deliver 2A7 across plasma membrane without fully saturating epitope-binding sites. Further optimization of this approach may result in more effective intracellular delivery of 2A7 to achieve sufficient intracellular antibody levels allowing for efficient blocking of HBx and HBx-DDB1 interaction.

In summary, results presented here provide 2A7 as a broadly reactive HBx monoclonal antibody capable of interfering with its interaction with a key cellular adapter protein DDB1, which is crucial for HBV life cycle. This suggests a high potential for 2A7 in HBx-targeting diagnostic and therapeutic applications. Availability of the full sequences of 2A7 and demonstration of the functional equivalence of recombinant 2A7 and 2A7-derived scFv compared to original 2A7 also paved way for further engineering of 2A7 for such applications.

## Methods

### Monoclonal antibodies and cells

Mouse anti-HBx mAb 2A7 prepared using bacterially expressed HBx has been described^27^. A different anti-HBx mAb designated 2A2 was obtained in parallel with 2A7. 2A7- and 2A2-producing SP2/0 hybridoma cell lines were maintained in RPMI 1640 medium (Invitrogen, China) supplemented with 15% fetal bovine serum at 37 °C with 5% CO_2_.

Human embryonic kidney cell line HEK293T and human hepatocellular carcinoma cell lines HepG2 and Huh7 were cultured in Dulbecco’s modified Eagle’s medium (Invitrogen, China) supplemented with 10% fetal bovine serum at 37 °C with 5% CO_2_. Expi293F^TM^ cells were grown in serum-free Expi293^TM^ Expression Medium (Invitrogen, China) at 37 °C with 8% CO_2_ on an orbital shaker platform (Thermo Scientific, China) rotating at 125 rpm.

Polyethylenimine (Sigma-Aldrich, China), ExpiFectamine™ 293 Reagent (Thermo Scientific, China) and TurboFect transfection reagent (Thermo Scientific, China) were used for transfecting HEK293T, Expi293F^TM^, and Huh7 and HepG2 cells, respectively, according to manufacturers’ instructions.

### Plasmids, primers, proteins and peptides

To construct FLAG-tagged HBx expression plasmids, X ORFs were amplified from HBV replicon plasmids of three genotype B strains (designated BPS, B6 and B200, respectively^34^) and one genotype C strain (designated WT^35^) using PCR and inserted downstream of FLAG tag in pcDNA3.0 vector (Invitrogen, China). Expression plasmids for HBx mutants were generated from wild type expression plasmids using KOD-plus mutagenesis kit (TOYOBO, China). Sequences of primers used from generating these HBx constructs are listed in Supplementary Table S1. Plasmids expressing untagged DDB1 (GenBank Accession NM_001923) and Cullin4A (GenBank Accession NM_001354943) were constructed by amplifying the ORFs from respective cDNA plasmids (Promega, China) and cloning the amplicons into pcDNA3.0. HA-tagged constructs were then generated using KOD-plus mutagenesis kit. Sequences of primers used for generating DDB1 and Cullin4A constructs are listed in Supplementary Table S2 & 3.

Bacterially expressed BPS-encoded HBx protein purified under denaturing conditions and used as immunogen for mAb generation has been described^27^. Series of biotinylated 16 amino acid peptides with 8 serially overlapping residues that altogether encompass full-length BPS-encoded HBx were chemically synthesized (GL Biochem, Shanghai, China). HBx fragment (a.a. 81–96) harboring 2A7 epitope with or without a.a. 47–58 of HIV-1 Tat protein at the C-terminal joined through an miniPEG3 linker, and control HBx peptides (a.a. 65–80, a.a. 73–88) were similarly synthesized.

### mAb cDNA cloning, sequencing and sequence analysis

Total RNA was extracted from mAb-producing hybridoma cells and subjected to RT-PCR using primer sets designed for amplifying mouse IgG constant and variable regions^30^ as listed in Supplementary Table S4–6. Amplicons with expected length were recovered from agarose gels and cloned into TA-vector (TaKaRa). Multiple randomly selected clones were then sequenced to obtain candidate IgG cDNA sequences. CDR within heavy and light chain variable regions was predicted using Vbase2 (www.vbase2.org).

### Mass spectrometry

Purified mAb proteins were analyzed by 12% SDS-PAGE under reducing conditions, followed by Coomassie Brilliant Blue G-250 (Sigma) staining. Bands corresponding to heavy and light chains of IgG were identified by apparent molecular weights and excised using clean scalpels. In-gel trypsin digestion, extraction of tryptic peptides and LC-MS/MS analyses were performed essentially as described previously^36^. LC-MS/MS and raw data collection was conducted by the core facility of Institutes of Biomedical Sciences, Shanghai Medical College, Fudan University on a hybrid quadrupole Orbitrap (Q-Exactive) mass spectrometer (Thermo Fisher Scientific, Bremen, Germany) coupled to a Nano Aquity UPLC system (Waters Corporation, Milford, MA). Machine-generated raw MS data were fed into MaxQuant^37^ software for protein identification using candidate IgG cDNA sequences as queries with default settings, along with commonly occurring protein contaminants.

### Recombinant antibody and scFv

Human-codon-optimized 2A7 heavy and light chain variable region coding sequences with N-terminal human IgG2 heavy chain secretion signal sequence and C-terminal mouse IgG2a heavy chain or κ light chain constant fragments, respectively, were synthesized and cloned into pcDNA3.4 vector (Invitrogen, China). To produce recombinant 2A7 antibody, Expi293F^TM^ cells were transfected with the obtained recombinant 2A7 heavy and light chain expression plasmids at 1:2 ratio, and culture supernatant was collected at 6 days post transfection, followed by centrifugation at 5000 *g* for 30 min and filtration through 0.22 μm filter. Filtrate was loaded onto RoboColumn holding Protein A affinity chromatography resin (Merck Millipore, China). After washing with PBS, bound recombinant antibody was eluted with 0.1 mol/L glycine (pH 2.6) into 1 mol/L Tris-HCl (pH 8.8).

2A7-derived scFv expression plasmids Igκ-VLVH-Fc and Igκ-VHVL-Fc were constructed by joining 2A7 heavy chain and light chain variable regions in reciprocal order with an intervening (GlyGlyGlyGlySer)_3_ linker through overlap extension PCR with primers listed in Supplementary Table S7. Amplicons were inserted into a modified pSecTag2A vector between N-terminal mouse Igκ secretion signal sequence and C-terminal human IgG1 Fc fragment (kindly provided by Prof. Tianlei Ying, Fudan University). The secretion signal sequences were removed using KOD-plus mutagenesis kit (TOYOBO) to generate plasmids VLVH-Fc and VHVL-Fc. To produce scFv, HEK293T cells were transfected with corresponding plasmid and 48 hours later, supernatants were harvested and cells were lysed with RIPA buffer (Thermo Scientific, China). For enrichment of scFv, supernatants or cell lysates were mixed with Protein A/G agarose (Santa Cruz, China) and incubated with rotation at 4 °C for 2 hours. Gels were washed 3 times with PBS and bound recombinant scFv was eluted with 0.1 mol/L glycine (pH 2.6) into 1 mol/L Tris-HCl (pH 8.8).

### ELISA, immunofluorescence and Western blot

Recombinant HBx protein was used for coating 96-well microplates at 100 ng/well in bicarbonate/carbonate coating buffer (50 mmol/L, pH9.6). For epitope mapping, biotinylated HBx peptides were added to streptavidin coated StreptaWell microplate (Roche, China) at 500 ng/well in PBS. Coating was performed at room temperature for 30 min and plates were then washed with 0.05% Tween-80 in PBS (PBST) and blocked with 3% bovine serum albumin (BSA) in PBS. Antibodies, cell supernatants or lysates, diluted in blocking buffer if necessary, were then added and incubated at 37 °C for 1 h, followed by washing and reaction with horseradish peroxidase (HRP)-conjugated anti-mouse pAb (Sigma-Aldrich, China) or anti-human Fc pAb (Beyotime, China). HRP substrates were then added and optical density at 450 nm (OD_450_) was measured after the addition of 0.1 mol/L HCl using a microplate reader (BioRad, China). Immunofluorescence and Western blot analyses were performed according to standard procedures as previously described^27,38^. Densitometry scanning was performed using ImageJ software.

### Co-immunoprecipitation and pull-down assays

Anti-FLAG M2 magnetic beads (Sigma Aldrich, China) and Protein A/G agarose (Santa Cruz, China) were used for capturing FLAG-tagged and Fc-containing proteins respectively in co-immunoprecipitation and pull-down assay. Cell lysates were prepared using IP lysis buffer (Thermo scientific, China) containing protease inhibitor cocktail (Thermo scientific, China) and mixed with beads. After incubation with rotation at 4 °C for 2 hours, beads were washed 4 times with PBST and then mixed with 1/3 volume of 4 × SDS sample buffer (0.2 mol/L Tris-HCl (pH 6.8), 8% SDS, 0.4 mol/L dithiothreitol, 40% glycerol, and 0.4% bromophenol blue) and heated at 95 °C for 3 minutes to elute the proteins. For pull-down assay with antibody blocking, cell lysates containing HA-tagged DDB1 and HA-tagged Cullin4A were first mixed with or without 2A7 or 2A2 (2 μg/ml), and then mixed with cell lysates containing FLAG-tagged HBx or HBx mutants and incubated with rotation at 4 °C for 2 hours before addition of anti-FLAG beads.

### Peptide-assisted cellular entry of antibody

2A7 mAb was mixed with different concentration of HBx peptide harboring 2A7 epitope fused with cell-penetrating peptide from HIV-1 Tat protein, incubated at 37 °C for 30 minutes and added to cell culture media. Cells were further cultured for 6 hours, washed 3 times with PBS, harvested following 0.25% trypsin/EDTA digestion and then washed twice with PBS. Harvested cells were lysed in SDS-PAGE loading buffer and analyzed for intracellular 2A7 mAb using Western blot, or lysed in RIPA buffer and analyzed in ELISA. As control, cells were also collected without trypsinization by washing 3 times with PBS and lysing in SDS-PAGE loading buffer. In order to demonstrate specificity of cellular entry enabled by the fusion peptide, HBx peptide harboring 2A7 epitope or neighboring fragment not encompassing 2A7 epitope was added during incubation, or mAb 2A2 was used in place of 2A7.

### HBx sequence retrieval and analysis

A total of 13950 HBx protein sequences were retrieved from GenBank in December, 2016, from which sequences with insertion or deletion, or not beginning with methionine were excluded, and the remaining 7098 full-length (154 a.a.) HBx sequences were obtained for analysis.

## Supplementary information

Supplementary Figures and Tables 1–7.

Supplementary Table 8.

## Data Availability

The datasets generated and analyzed in the current study are available from the corresponding author on reasonable request.
