# Supplementary material for: Characterization and engineering of broadly reactive monoclonal antibody against hepatitis B virus X protein that blocks its interaction with DDB1
Source: Sci Rep. 2019 Dec 30;9:20323. doi: 10.1038/s41598-019-56819-8 (PMC6937242; doi:10.1038/s41598-019-56819-8)
Supplement: Supplementary file 1 — Supplementary Figures and Tables 1–7. [file 41598_2019_56819_MOESM1_ESM.pdf]

Supplementary Information

Characterization and engineering of broadly reactive monoclonal antibody against hepatitis B virus X protein that blocks its interaction with DDB1

Shuai Tao, Shaokun Pan, Chenjian Gu, Lili Wei, Ning Kang, Youhua Xie and Jing Liu

Supplementary Figure S1~S6

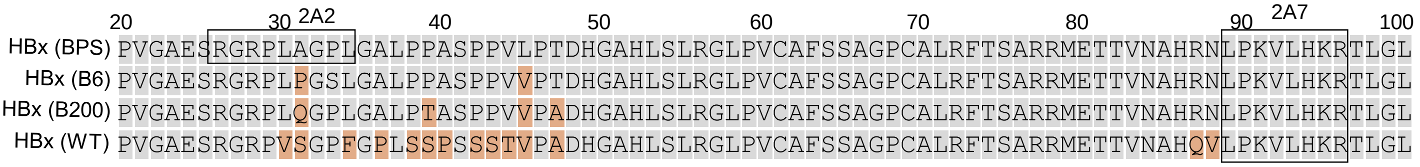

Supplementary Figure S1. Protein sequence (partial) alignment of HBx encoded by genotype B and C HBV strains used in this work.

HBx encoding sequences of genotype B strains BPS, B6 and B200, and genotype C strain WT are aligned with the 2A7 and 2A2 epitope indicated using open rectangles. Colored residues indicate difference from BPS HBx.

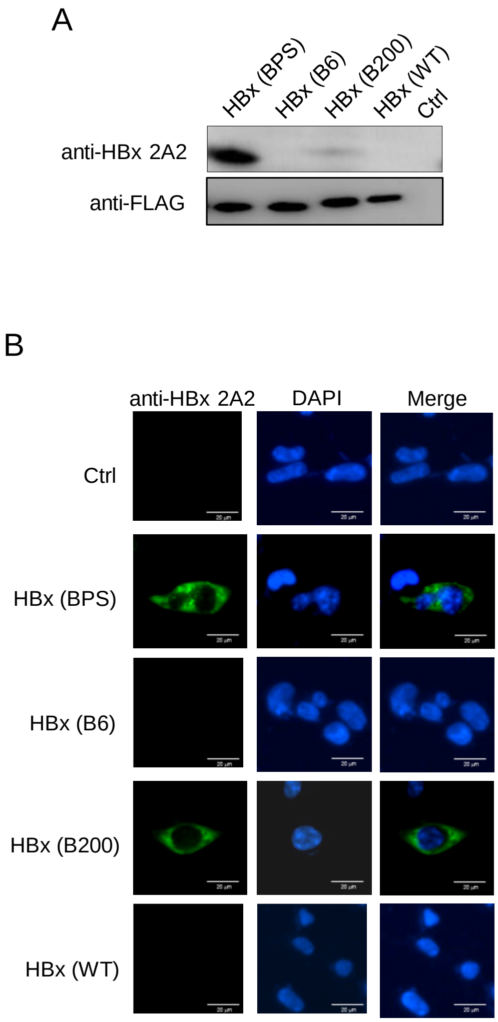

Supplementary Figure S2. Anti-HBx mAb 2A2 reacts with native and denatured HBx encoded by limited HBV strains

HEK293T cells were transfected with plasmids expressing indicated HBx variants fused to N-terminal FLAG tag, and 48 hours later, were analyzed in Western blot (**A**) and immunofluorescence (**B**) using monoclonal anti-HBx antibody 2A2. FLAG antibody was used as control in Western blot (A). Cell nuclei were stained using DAPI (B). Scale bars, 20 μm.

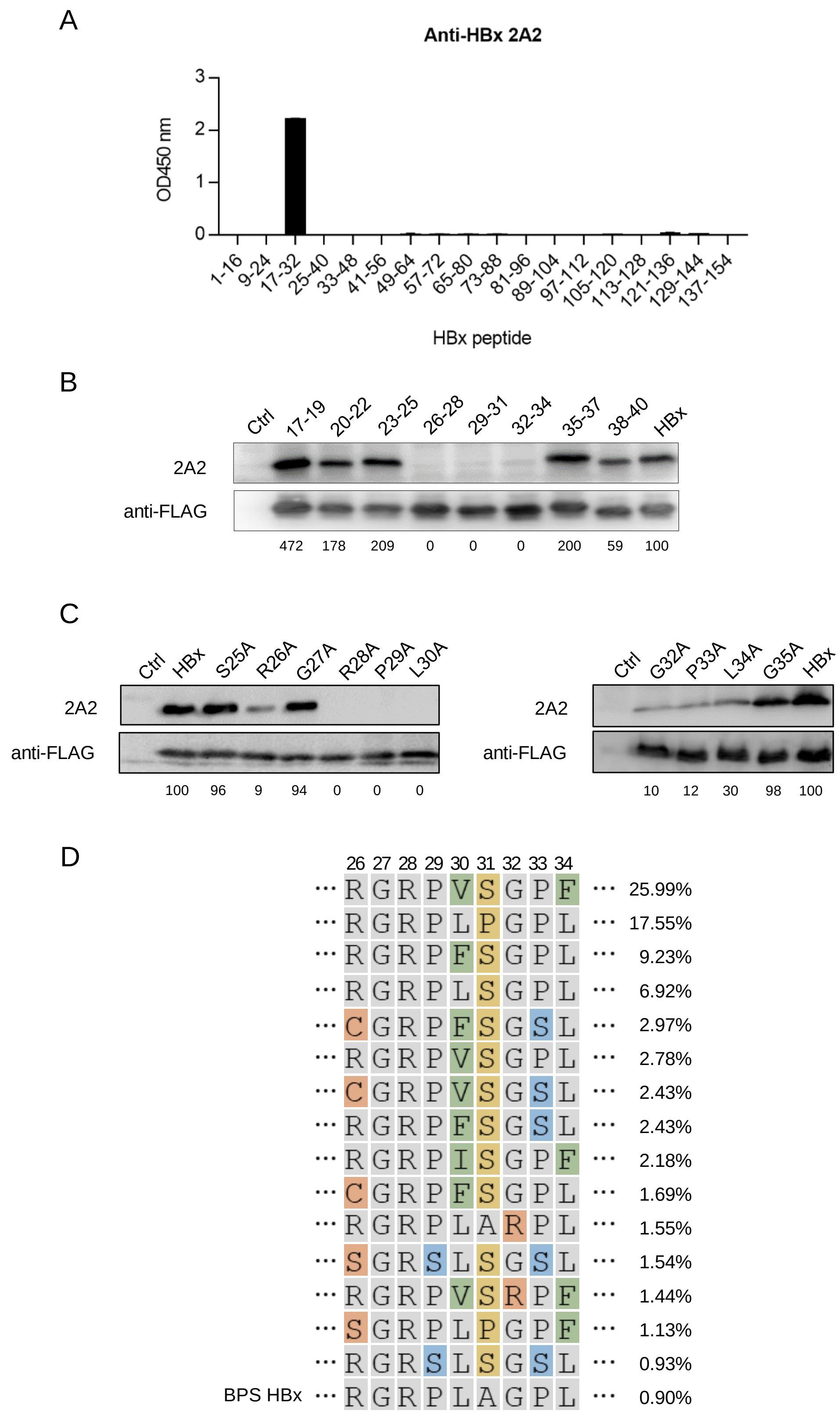

Supplementary Figure S3. Epitope analysis of anti-HBx mAb 2A2.

(**A**) Serially overlapping biotinylated peptides encompassing full BPS HBx length were captured onto Streptavidin-coated plates and subjected to ELISA using 2A2. Plasmids expressing FLAG-tagged BPS HBx with serial 3 amino acid residues mutated to 3 Ala (**B**) , or indicated single residue mutations (**C**), were transfected into HEK293T. Transfected cells were analyzed in Western blot using 2A2 and FLAG antibody. After densitometry scanning, 2A2-generated signals were first adjusted against anti-FLAG-generated signals, followed by normalization against BPS HBx. Normalized values are indicated as percentages. (**D**) Alignment of most frequently found types of sequences at 2A2 epitope (a.a. 26-34) with Colored residues indicating difference from BPS HBx.

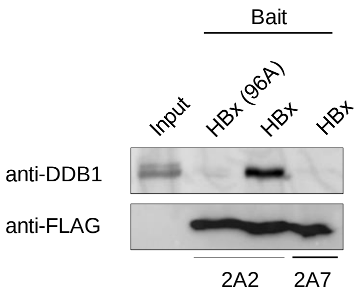

Supplementary Figure S4. Anti-HBx mAb 2A2 does not interfere with HBx-DDB1 interaction

HEK293T cells transfected with DDB1 and cells transfected with wild type or R96A mutant BPS HBx expression plasmids as indicated were used to prepare cell lysates. DDB1 containing lysates were then mixed with FLAG-HBx-containing lysates with addition of 2A7 or 2A2 antibody, and subjected to pulldown using anti-FLAG antibody. Captured proteins were analyzed using anti-FLAG and anti-DDB1 antibodies.

2A7 VH (**MHV.B4/MHC**):

CAGGTTACTCTGAAAGAGTCTGGCCCTGGGATATTGCAGCCCTCCCAGACCCTCAGTCTGACTTGTTCTTTC
TCTGGGTTTTCACTGAGCACTTCTGGTATGGGTGTAGTCTGGATTCGTCAGCCTTCAGGGAAGGGTCTGGAG
TGGCTGGCACACATTTGGTGGGATGATGATAAGTACTATAACACAGCCCTGAAGAGCGGGCTCACAATCTCC
AAGGATACGTCCAAAAACCAGGTCTTCCTCAAGATCGCCAGTGTGGACACTGCAGATACTGCCACATACTAC
TGTGCTCGAATGCCCTACTATGATTACGACGGGGGGGTTGCTTACTGGGGCCAAGGGACTCTGGTCACTGTC
TCTGCA**GCCAAAACGACACCCCCATCTGTCTATCCACTGGCC**

QVTLKESGPGILQPSQTLSLTCSFSGFSLSTSGMGVVWIRQPSGKGLEWLAHIWWDDDKYYNTALKSGLTIS

CDR1 CDR2

KDTSKNQVFLKIASVDTADTATYYCARMPYYDYDGGVAYWGQGTLVTVSA**AKTTPPSVYPLA**

CDR3

2A7 VL1 (**MKV.B5/MKC**):

GACATTGTGATGACCCAGTCTCCTGCTTCCTTAGTTGTATCTCTGGGGCAGAGGGCCACCATCTCATGCAGG
GCCAGCAAAAGTGTCAGTACATCTGGCTATAGTTATATGTACTGGTACCAACAGAAACCAGGACAGCCACCC
AAACTCCTCATCTATCTTGCATCCAACCTAGAATCTGGGGTCCCTGCCAGGTTCAGTGGCAGTGGGTCTGGG
ACAGACTTCACCCTCAACATCCATCCTGTGGAGGAGGAGGATGCTGCAACCTATTACTGTCAGCACAGTAGG
GAGCTTCCGCTCACGTTCGGTGCTGGGACCAAGCTGGAGCTGAAACGGGCT**GATGCTGCACCAACTGTATCC**

DIVMTQSPASLVVSLGQRATISCRASKSVSTSGYSYMYWYQQKPGQPPKLLIYLASNLESGVPARFSGSGSG

CDR1 CDR2

TDFTLNIHPVEEEDAATYYCQHSRELPLTFGAGTKLELKRA**DAAPTVS**

CDR3

2A7 VL2 (**MKV.B5/MKC**):

GACATTGTGATGACCCAGTCTCAAAAATTCATGTCCACATCAGTAGGAGACAGGGTCGGCACCACC**TAA**AAG
GCCAGTCAGAATGTTCGTACTGCCGTAGCCTGGTATCAACAGAAACCAGGGCAGTCTCCTAAAGCACTGATT
TACTTGGCATCAAACCGGCACACTGGAGTCCCTGATCGCTTCACGGGCAGAGGATCTGGGACAGATTTCACT
CTCACCATTAGCAATGTGCAATCTGAAGACCTGGCAGATTATTTCTGTCTGCAACATTGGAATTATCCTCAC
ACGTTCGGAGGGGGGACCAAGCTGGAAATAAAACGGGCT**GATGCTGCACCAACTGTATCC**

DIVMTQSQKFMSTSVGDRVGTT*KASQNVRTAVAWYQQKPGQSPKALIYLASNRHTGVPDRFTGRGSGTDFT
LTISNVQSEDLADYFCLQHWNYPHTFGGGTKLEIKRA**DAAPTVS**

Supplementary Figure S5. IgG variable region cDNA sequences amplified from 2A7 hybridoma cells

Primer sets used for amplification are specified in brackets. Nucleic acid and corresponding protein sequences are listed, with those derived from primers in bold. Internal stop codon in 2A7 VL2 is highlighted in red. Predicted CDR regions within 2A7 VH and VL1 protein sequences are indicated.

2A7 CH (**MHC.F/MHC.R**):

CAAAACGACACCCCCATCTGTCTATCCACTGGCCCCTGGATCTGCTGCCCAAACTAACTCCATGGTGACCCTGGGATG
CCTGGTCAAGGGCTATTTCCCTGAGCCAGTGACAGTGACCTGGAACTCTGGATCCCTGTCCAGCGGTGTGCACACCTT
CCCAGCTGTCCTGCAGTCTGACCTCTACACTCTGAGCAGCTCAGTGACTGTCCCCTCCAGCACCTGGCCCAGCGAGAC
CGTCACCTGCAACGTTGCCCACCCGGCCAGCAGCACCAAGGTGGACAAGAAAATTGTGCCCAGGGATTGTGGTTGTAA
GCCTTGCATATGTACAGTCCCAGAAGTATCATCTGTCTTCATCTTCCCCCCAAAGCCCAAGGATGTGCTCACCATTAC
TCTGACTCCTAAGGTCACGTGTGTTGTGGTAGACATCAGCAAGGATGATCCCGAGGTCCAGTTCAGCTGGTTTGTAGA
TGATGTGGAGGTGCACACAGCTCAGACGCAACCCCGGGAGGAGCAGTTCAACAGCACTTTCCGCTCAGTCAGTGAACT
TCCCATCATGCACCAGGACTGGCTCAATGGCAAGGAGTTCAAATGCAGGGTCAACAGTGCAGCTTTCCCTGCCCCCAT
CGAGAAAACCATCTCCAAAACCAAAGGCAGACCGAAGGCTCCACAGGTGTACACCATTCCACCTCCCAAGGAGCAGAT
GGCCAAGGATAAAGTCAGTCTGACCTGCATGATAACAGACTTCTTCCCTGAAGACATTACTGTGGAGTGGCAGTGGAA
TGGGCAGCCAGCGGAGAACTACAAGAACACTCAGCCCATCATGGACACAGATGGCTCTTACTTCGTCTACAGCAAGCT
CAATGTGCAGAAGAGCAACTGGGAGGCAGGAAATACTTTCACCTGCTCTGTGTTACATGAGGGCCTGCACAACCACCA
TACTGAGAAG**AGCCTCTCCCACTCTCCTGGTAAA**

KTTPPSVYPLAPGSAAQTNSMVTLGCLVKGYFPEPVTVTWNSGSLSSGVHTFPAVLQSDLYTLSSSVTVPSSTWPSET
VTCNVAHPASSTKVDKKIVPRDCGCKPCICTVPEVSSVFIFPPKPKDVLTITLTPKVTCVVVDISKDDPEVQFSWFVD
DVEVHTAQTQPREEQFNSTFRSVSELPIMHQDWLNGKEFKCRVNSAAFPAPIEKTISKTKGRPKAPQVYTIPPPKEQM
AKDKVSLTCMITDFFPEDITVEWQWNGQPAENYKNTQPIMDTDGSYFVYSKLNVQKSNWEAGNTFTCSVLHEGLHNHH
TEK**SLSHSPGK**

2A7 CK (**MKC.F/MKC.R**)

CGGGCTGATGCTGCACCAACTGTATCCATCTTCCCACCATCCAGTGAGCAGTTAACATCTGGAGGTGCCTCAGTCGTG
TGCTTCTTGAACAACTTCTACCCCAAAGACATCAATGTCAAGTGGAAGATTGATGGCAGTGAACGACAAAATGGCGTC
CTGAACAGTTGGACTGATCAGGACAGCAAAGACAGCACCTACAGCATGAGCAGCACCCTCACGTTGACCAAGGACGAG
TATGAACGACATAACAGCTATACCTGTGAGGCCACTCACAAGACATCAACTTCACCCATTGTC**AAGAGCTTCAACAGG
AATGAGTGTTAG**

RADAAPTVSIFPPSSEQLTSGGASVVCFLNNFYPKDINVKWKIDGSERQNGVLNSWTDQDSKDSTYSMSSTLTLTKDE
YERHNSYTCEATHKTSTSPIV**KSFNRNEC**

2A7 CL1 (**MLC.F/MLC.R**)

GTTTATTTTCGGCAGTGGAACCAAGGTCACTGTCCTAGGTCAGCCCAAGTCCACTCCCACACTCACCATGTTTCCACC
TTCCCCTGAGGAGCTCCAGGAAAACAAAGCCACACTCGTGTGTCTGATTTCCAATTTTTCCCCAAGTGGTGTGACAGT
GGCCTGGAAGGCAAATGGTACACCTATCACCCAGGGTGTGGACACTTCAAATCCCACCAAAGAGGACAACAAGTACAT
GGCCAGCAGCTTCTTACATTTGACATCGGACCAGTGGAGATCTCACAACAGTTTTACC**TGCCAAGTTACACATGAAGG**

FIFGSGTKVTVLGQPKSTPTLTMFPPSPEELQENKATLVCLISNFSPSGVTVAWKANGTPITQGVDTSNPTKEDNKYM
ASSFLHLTSDQWRSHNSFT**CQVTHE**

2A7 CL2 (**MLC.F/MLC.R**)

GTTTATTTTCGGCAGTGGAACCAAGGTCACTGTCCTAGGTCAGCCCAAGTCCACTCCCACTCTCACCATGTTTCCACC
TTCCTCTGAGGAGCTCCAGGAAAACAAAGCCACACTGGTGTGTCTGATTTCCAATTTTTCCCCAAGTGGTGTGACAGT
GGCCTGGAAGGCAAATGGTACACCTATCACCCAGGGTGTGGACACTTCAAATCCCACCAAAGAGGACAACAAGTACAT
GGCCAGCAGCTTCTTACATTTGACATCGGACCAGTGGAGATCTCACAACAGTTTTACC**TGCCAAGTTACACATGAAGG**

FIFGSGTKVTVLGQPKSTPTLTMFPPSSEELQENKATLVCLISNFSPSGVTVAWKANGTPITQGVDTSNPTKEDNKYM
ASSFLHLTSDQWRSHNSFT**CQVTHE**

Supplementary Figure S6. IgG constant region cDNA sequences amplified from 2A7 hybridoma cells

Primer sets used for amplification are specified in brackets. Nucleic acid and corresponding protein sequences are listed, with those derived from primers in bold.

Supplementary Table S1~S7

Supplementary Table S1. Primers for constructing FLAG-tagged HBx and HBx mutant expression plasmids

Primer	Primer sequence (5’-3’)	HBx variant
HBx-F	TAAAATCGATATGGCTGCTAGGGTGTGCTG	HBx (BPS)<br>HBx (B6)
HBx-R	CATGCTCGAGTTAGGCAGAGGTGAAAAAGTT	HBx (B200)<br>HBx (wt)
HBx 17-19 F	**CCGCC**CCCGTCGGCGCTGAATCCCGCGGA	HBx (17-19)
HBx 17-19 R	**CGGC**AAGGACGTCCCGCGCAGGATCCAG	
HBx 20-22 F	**CCGCC**GCTGAATCCCGCGGACGACCCCTC	HBx (20-22)
HBx 20-22 R	**CGGC**ACGTAAACAAAGGACGTCCCGCGC	
HBx 23-25 F	**CCGCC**CGCGGACGACCCCTCCAGGGGCCG	HBx (23-25)
HBx 23-25 R	**CGGC**GCCGACGGGACGTAAACAAAGGAC	
HBx 26-28 F	**CCGCC**CCCCTCCAGGGGCCGCTTGGGGCT	HBx (26-28)
HBx 26-28 R	**CGGC**GGATTCAGCGCCGACGGGACGTAA	
HBx 29-31 F	**CCGCC**GGGCCGCTTGGGGCTCTACCGACC	HBx (29-31)
HBx 29-31 R	**CGGC**TCGTCCGCGGGATTCAGCGCCGAC	
HBx 32-34 F	**CCGCC**GGGGCTCTACCGACCGCTTCTCCG	HBx (32-34)
HBx 32-34 R	**CGGC**CTGGAGGGGTCGTCCGCGGGATTC	
HBx 35-37 F	**CCGCC**CCGACCGCTTCTCCGCCTGTTGTA	HBx (35-37)
HBx 35-37 R	**CGGC**AAGCGGCCCCTGGAGGGGTCGTC	
HBx 38-40 F	**CCGCC**TCTCCGCCTGTTGTACCGGCCGAC	HBx (38-40)
HBx 38-40 R	**CGGC**TAGAGCCCCAAGCGGCCCCTGGAG	
HBx 77-79 F	**CCGCC**GAGACCACCGTGAACGCCCACCGG	HBx (77-79)
HBx 77-79 R	**CGGC**TGCAGAGGTGAAGCGAAGTGCACA	
HBx 80-82 F	**CCGCC**GTGAACGCCCACCGGAACTTGCCT	HBx (80-82)
HBx 80-82 R	**CGGC**CATGCGACGTGCAGAGGTGAAGCG	
HBx 83-85 F	**CCGCC**CACCGGAACTTGCCTAAGGTCTTG	HBx (83-85)
HBx 83-85 R	**CGGC**GGTGGTCTCCATGCGACGTGCAGA	
HBx 86-88 F	**CCGCC**TTGCCTAAGGTCTTGCATAAGAGG	HBx (86-88)
HBx 86-88 R	**CGGC**GGCGTTCACGGTGGTCTCCATGCG	
HBx 89-91 F	**CCGCC**GTCTTGCATAAGAGGACTCTTGGA	HBx (89-91)
HBx 89-91 R	**CGGC**GTTCCGGTGGGCGTTCACGGTGGT	
HBx 92-94 F	**CCGCC**AAGAGGACTCTTGGACTTTCAGCA	HBx (92-94)
HBx 92-94 R	**CGGC**CTTAGGCAAGTTCCGGTGGGCGTT	
HBx 95-97 F	**CCGCC**CTTGGACTTTCAGCAATGTCAACG	HBx (95-97)
HBx 95-97 R	**CGGC**ATGCAAGACCTTAGGCAAGTTCCG	
HBx 98-100 F	**CCGCC**TCAGCAATGTCAACGACCGACCTT	HBx (98-100)
HBx 98-100	**CGGC**AGTCCTCTTATGCAAGACCTTAGG	

R		
HBx 101-103 F<br>HBx 101-103 R	**CCGCC**TCAACGACCGACCTTGAGGCATAC<br>**CGGC**AAGTCCAAGAGTCCTCTTATGCAA	HBx (101-103)
HBx 104-106 F<br>HBx 104-106 R	**CCGCC**GACCTTGAGGCATACTTCAAAGAC<br>**CGGC**CATTGCTGAAAGTCCAAGAGTCCT	HBx (104-106)
HBx 25 F<br>HBx 25 R	**CG**CGCGGACGACCCCTCGCGGG<br>**C**TTCAGCGCCGACGGGACGTA	HBx (S25A)
HBx 26 F<br>HBx 26 R	**CG**GGACGACCCCTCGCGGGGCC<br>**C**GGATTCAGCGCCGACGGGAC	HBx (R26A)
HBx 27 F<br>HBx 27 R	**CG**CGACCCCTCGCGGGGCCGCT<br>**C**GCGGGATTCAGCGCCGACGG	HBx (G27A)
HBx 28 F<br>HBx 28 R	**CG**CCCCTCGCGGGGCCGCTTGG<br>**C**TCCGCGGGATTCAGCGCCGA	HBx (R28A)
HBx 29 F<br>HBx 29 R	**CG**CTCGCGGGGCCGCTTGGGGC<br>**C**TCGTCCGCGGGATTCAGCGC	HBx (P29A)
HBx 30 F<br>HBx 30 R	**CG**GCGGGGCCGCTTGGGGCTCT<br>**C**GGGTCGTCCGCGGGATTCAG	HBx (L30A)
HBx 32 F<br>HBx 32 R	**CG**CCGCTTGGGGCTCTACCGCC<br>**C**CGCGAGGGGTCGTCCGCGGG	HBx (G32A)
HBx 33 F<br>HBx 33 R	**CG**CTTGGGGCTCTACCGCCCGC<br>**C**CCCCGCGAGGGGTCGTCCGC	HBx (P33A)
HBx 34 F<br>HBx 34 R	**CG**GGGGCTCTACCGCCCGCTTC<br>**C**CGGCCCCGCGAGGGGTCGTC	HBx (L34A)
HBx 35 F<br>HBx 35 R	**CG**GCTCTACCGCCCGCTTCTCC<br>**C**AAGCGGCCCCGCGAGGGGTC	HBx (G35A)
HBx 89 F<br>HBx 89 R	**CC**CCCCAAGGTCTTGCATAAGAG<br>**C**GTTCCGGTGGGCGTTCACGG	HBx (L89A)
HBx 90 F<br>HBx 90 R	**CC**AAGGTCTTGCATAAGAGGAC<br>**C**CAAGTTCCGGTGGGCGTTCA	HBx (P90A)
HBx 91 F<br>HBx 91 R	**CC**GTCTTGCATAAGAGGACTCT<br>**C**GGGCAAGTTCCGGTGGGCGT	HBx (K91A)
HBx 92 F<br>HBx 92 R	**CC**TTGCATAAGAGGACTCTTGG<br>**C**CTTGGGCAAGTTCCGGTGGG	HBx (V92A)
HBx 93 F<br>HBx 93 R	**CC**CATAAGAGGACTCTTGGACT<br>**C**GACCTTGGGCAAGTTCCGGT	HBx (L93A)
HBx 94 F	**CC**AAGAGGACTCTTGGACTTTC	HBx (H94A)

HBx 94 R	**C**CAAGACCTTGGGCAAGTTCC	
HBx 95 F	**CC**AGGACTCTTGGACTTTCAGC	HBx (K95A)
HBx 95 R	**C**ATGCAAGACCTTGGGCAAGT	
HBx 96 F	**CC**ACTCTTGGACTTTCAGCAAT	HBx (R96A)
HBx 96 R	**C**CTTATGCAAGACCTTGGGCA	
HBx 97 F	**CC**CTTGGACTTTCAGCAATGTC	HBx (T97A)
HBx 97 R	**C**CCTCTTATGCAAGACCTTGG	
HBx 98 F	**CC**GGACTTTCAGCAATGTCAAC	HBx (L98A)
HBx 98 R	**C**AGTCCTCTTATGCAAGACCT	
var1 F	**AC**AAGAGGACTCTTGGACTTTC	HBx (var1)
var1 R	**A**CAAGACCTTGGGCAAGTTCC	
var2 F	**CG**GTCTTGCATAAGAGGACTCT	HBx (var2)
var 2 R	**T**GGGCAAGTTCCGGTGGGCGT	
var 3 F	TTG**TAC**AAGAGGACTCTTGGACTTTC	HBx (var 3)
var 3 R	**TTGCCT**GGGCAAGTTCCGGTGGGCGT	
var 4 F	**TC**TTGCATAAGAGGACTCTTGG	HBx (var 4)
var 4 R	**T**CTTGGGCAAGTTCCGGTGGG	
var 5 F	**TT**TTGCATAAGAGGACTCTTGG	HBx (var 5)
var 5 R	**G**CTTGGGCAAGTTCCGGTGGG	
var 6 F	**AA**AGGACTCTTGGACTTTCAGC	HBx (var 6)
var 6 R	**G**ATGCAAGACCTTGGGCAAGT	

Bold bases represent mutations.

Supplementary Table S2. Primers for constructing HA-tagged DDB1 expression plasmids

Primer	Primer sequence (5'-3')	Plasmid
DDB1 F	TGCAGATATCATGTCGTACAACTACGTGGTA	pCMV-DDB1
DDB1 R	GCCCTCTAGACTAATGGATCCGAGTTAGCTC	
DDB1-HA F	**TCCCCGACTACGCC**TAGCCAAGGGCAGGGGGCCCCTTT	pCMV-DDB1-HA
DDB1-HA R	**CGTCGTAGGGGTA**ATGGATCCGAGTTAGCTCCTC	

Bold bases encode HA tag.

Supplementary Table S3. Primers for constructing HA-tagged Cullin4A expression plasmids

Primer	Primer sequence (5'-3')	Plasmid
Cullin4A F	CGCCGGATCCATGGCGGACGAGGCCCCGCGGA	pcDNA-Cullin4A
Cullin4A R	GCTGGATATCTCAGGCCACGTAGTGGTACTGAT	
HA-Cullin4A F	**CGTCCCCGACTACGCC**GGATCCATGGCGGACGAGGCC	pcDNA-N-HA-Cullin4A
HA-Cullin4A R	**TCGTAGGGGTATCCCAT**GGTAAGCTTGGGTCTCCCTAT	

Bold bases encode HA tag.

Supplementary Table S4. Primers for amplifying mouse IgG heavy chain variable region

Primer	Primer sequence (5’-3’)
MHC	GGCCAGTGGATAGACAGATGGGGGTGTCGTTTTGGC
MHV.B1	GATGTGAAGCTTCAGGAGTC
MHV.B2	CAGGTGCAGCTGAAGGAGTC
MHV.B3	CAGGTGCAGCTGAAGCAGTC
MHV.B4	AGGTTACTCTGAAAGAGTC
MHV.B5	GAGGTCCAGCTGCAACAATCT
MHV.B6	GAGGTCCAGCTGCAGCAGTC
MHV.B7	CAGGTCCAACTGCAGCAGCCT
MHV.B8	GAGGTGAAGCTGGTGGAGTC
MHV.B9	GAGGTGAAGCTGGTGGAATC
MHV.B10	GATGTGAACTTGGAAGTGTC
MHV.B12	GAGGTGCAGCTGGAGGAGTC

Supplementary Table S5. Primers for amplifying mouse IgG light chain variable region

Primer	Primer sequence (5'-3')
MKC	GGATACAGTTGGTGCAGCATC
MKV.B1	GATGTTTTGATGACCCAAACT
MKV.B2	GATATTGTGATGACGCAGGCT
MKV.B3	GATATTGTGATAACCCAG
MKV.B4	GACATTGTGCTGACCCAATCT
MKV.B5	GACATTGTGATGACCCAGTCT
MKV.B6	GATATTGTGCTAACTCAGTCT
MKV.B7	GATATCCAGATGACACAGACT
MKV.B8	GACATCCAGCTGACTCAGTCT
MKV.B9	CAAATTGTTCTCACCCAGTCT
MKV.B10	GACATTCTGATGACCCAGTCT
MLC	GGTGAGTGTGGGAGTGGACTTGGGCTG
MLV.B	CAGGCTGTTGTGACTCAGGAA

MKC/MKV and MLC/MLV primers target kappa and lambda light chains, respectively.

Supplementary Table S6. Primers for amplifying mouse IgG constant regions

Primer	Primer sequence (5’-3’)	Targeted constant region
MHC.F	CAAAACGACACCCCCATCTG	Heavy chain
MHC.R	TTTACCAGGAGAGTGGGAGAGG	
MKC.F	CGGGCTGATGCTGCAC	Kappa light chain
MKC.R	CTAACACTCATTCCTGTTGAAGCTC	
MLC.F	GTTTATTTTCGGCAGTGGAAC	Lamda light chain
MLC.R	CCTTCATGTGTAACTTGGCA	

Supplementary Table S7. Primers for constructing 2A7 ScFv expression plasmids

Primer	Primer sequence (5'-3')	Plasmid
VH F1	GCGAAGCTTCAGGTTACTCTGAAAGAGT	IgκVHVL-Fc
VH R1	**ACCACCAGATCCACCTCCACCCGAGCC**ACCGCCACCTGGGGGTGTCGTTTTGGC	
VL F1	**GGCTCGGGTGGAGGTGGATCTGGT**GGTGGCGGTTCGGACATTGTGATGACCCAGTC	
VL R1	TGTCGGATCCGGATACAGTTGGTGCAGCAT	
VL F2	GCGAAGCTTGACATTGTGATGACCCAGTCT	IgκVLVH-Fc
VL R2	**ACCACCAGATCCACCTCCACCCGAGCC**ACCGCCACCGGATACAGTTGGTGCAGCATCAGCCC	
VH F2	**GGCTCGGGTGGAGGTGGATCTGGT**GGTGGCGGTTCGCAGGTTACTCTGAAAGAGTCTG	
VH R2	GTCGGATCCTGGGGGTGTCGTTTTGGCTG	
VH F3	AGGTTACTCTGAAAGAGTC	VHVL-Fc
VH R3	CATGGTGGCTAGCCAGCTTGGGTCT	
VL F3	GACATTGTGCTGACCCAATCT	VLVH-Fc
VL R3	CATGGTGGCTAGCCAGCTTGGGTCT	

Bold bases represent overlapping region between VH and VL fragments.
